# Saprophytic Pneumococci

**Published:** 1898-05-07

**Authors:** 


### SAPROPHYTIC PNEUMOCOCCI.

The results of certain researches as to the frequency with which pneumococci are to be found on the surface of the tonsil have recently been reported to the Medical Society of the Hospitals in Paris, and they show that these cocci are of far more constant occurrence than has usually been imagined. It had previously been stated that they could be found in about one-fifth of the healthy throats examined, but these researches indicated that the pneumococcus may be present in a far larger proportion of cases, having, in fact, been constantly found existin gas a saprophyte, in the same way as the streptococcus, in the forty healthy persons who were examined. The importance of the *rôle* played by the pneumococcus in the production of disease is thus greatly lessened. Pneumonia, no doubt, is an infective disease, but it appears that it is set up, not by exposure to an infection, but by exposure to those conditions which enable the infection (which, indeed, a man may carry about with him all the time) to take root, and permit the cocci to develop in parts where they can do harm. When a man gets "wet through," or "chilled to the bone," that is quite as important a factor in the production of the consequent pneumonia as is the pneumococcus, essential as the latter may be.

